# Photosynthetic control at the cytochrome *b*_6_*f* complex

**DOI:** 10.1093/plcell/koae133

**Published:** 2024-04-26

**Authors:** Gustaf E Degen, Matthew P Johnson

**Affiliations:** Plants, Photosynthesis and Soil, School of Biosciences, University of Sheffield, Sheffield S10 2TN, UK; Plants, Photosynthesis and Soil, School of Biosciences, University of Sheffield, Sheffield S10 2TN, UK

## Abstract

Photosynthetic control (PCON) is a protective mechanism that prevents light-induced damage to PSI by ensuring the rate of NADPH and ATP production via linear electron transfer (LET) is balanced by their consumption in the CO_2_ fixation reactions. Protection of PSI is a priority for plants since they lack a dedicated rapid-repair cycle for this complex, meaning that any damage leads to prolonged photoinhibition and decreased growth. The imbalance between LET and the CO_2_ fixation reactions is sensed at the level of the transthylakoid ΔpH, which increases when light is in excess. The canonical mechanism of PCON involves feedback control by ΔpH on the plastoquinol oxidation step of LET at cytochrome *b*_6_*f*. PCON thereby maintains the PSI special pair chlorophylls (P700) in an oxidized state, which allows excess electrons unused in the CO_2_ fixation reactions to be safely quenched via charge recombination. In this review we focus on angiosperms, consider how photo-oxidative damage to PSI comes about, explore the consequences of PSI photoinhibition on photosynthesis and growth, discuss recent progress in understanding PCON regulation, and finally consider the prospects for its future manipulation in crop plants to improve photosynthetic efficiency.

## Introduction

Photosynthesis involves the production of the metabolites ATP and NADPH via light-powered coupled electron-proton transfer reactions across the chloroplast thylakoid membrane. These metabolites then power the fixation of CO_2_ into carbohydrates in the stroma (dark reactions or Calvin-Benson-Bassham [CBB] cycle reactions). Since light cannot be stored by photosynthetic organisms, they must carefully balance the production and consumption of these metabolites. A mismatch in the rate of light and CBB cycle reactions can result in the formation of reactive oxygen species (ROS) and cellular damage in excess light or underperformance and limitation on growth in limiting light (Li et al. 2009; Foyer et al. 2012). Balance is achieved via a plethora of feedforward and feedback regulatory control mechanisms that modulate the activity of the various photosynthetic enzymes (Buchanan 2016; Gurrieri et al. 2021). A crucial mechanism for the regulation of the light reactions is photosynthetic control (PCON), which involves feedback regulation of the electron-proton transfer reactions by high ΔpH under conditions where NADPH and ATP are in excess of that required for CO_2_ fixation (Foyer et al. 1990; Colombo et al. 2016). PCON protects PSI from light-induced damage by regulating the rate of electron transfer (Rumberg and Siggel 1969). Experiments in fluctuating light environments demonstrate the necessity of PCON for plant growth and resilience, with mutants lacking the process suffering severe damage to PSI (Suorsa et al. 2012).

**Figure 1.**
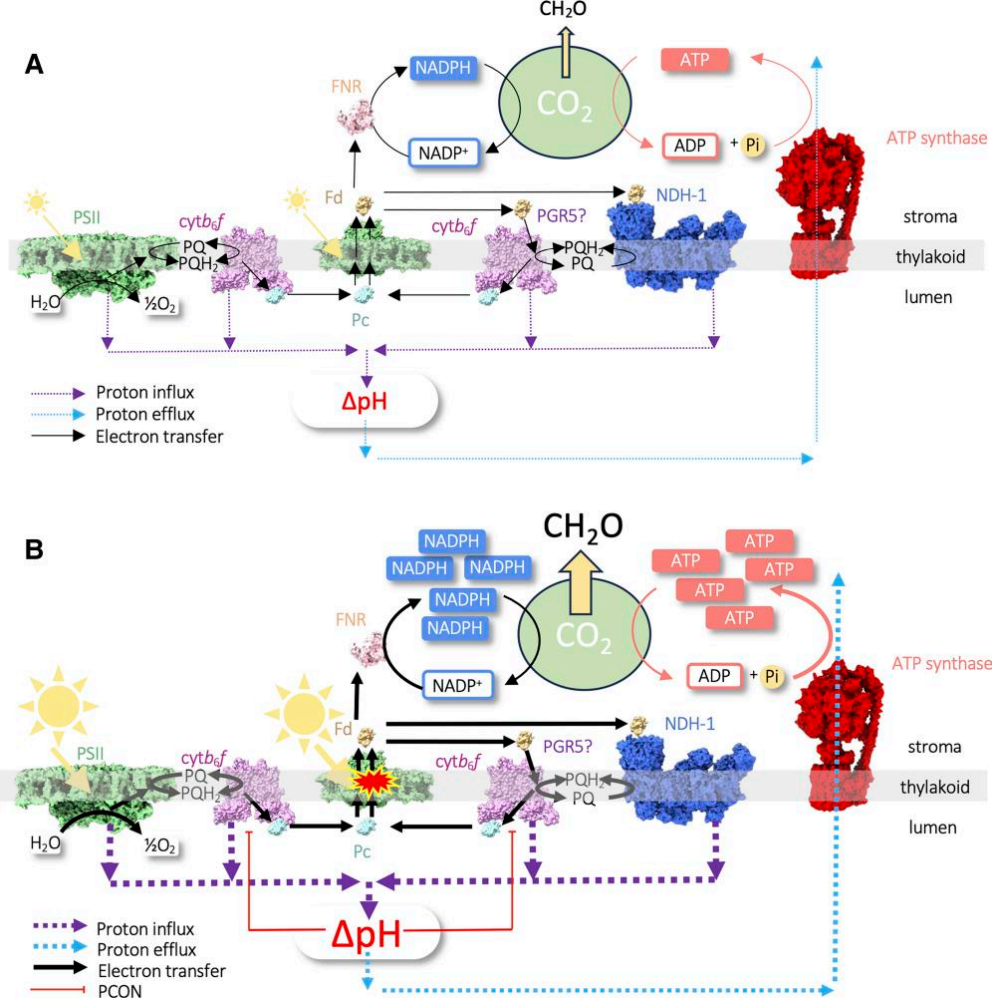
Regulation of the photosynthetic electron transport chain. **A)** In low-light conditions LET occurs from H_2_O via PSII, PQ, cyt*b*_6_*f*, Pc, PSI, Fd, and FNR to NADP^+^ forming NADPH. Protons are deposited into the lumen at PSII and cyt*b*_6_*f* driving ΔpH formation. ΔpH drives proton extrusion through ATP synthase to form ATP. Cyclic electron transfer (CET) contributes to ΔpH formation and ATP synthesis via 2 routes involving the NDH-1 and PGR5 pathways (Table 1). NADPH and ATP production are balanced by their consumption during CO_2_ fixation into carbohydrate (CH_2_O). **B)** In excess-light conditions, the production of NADPH and ATP exceeds their consumption in CO_2_ fixation; this leads to an overreduction of the electron transfer chain and photo-oxidative damage by ROS at PSI (red explosion). This damage is mitigated by the slower consumption of ATP, which ensures proton influx exceeds efflux from the lumen, building up ΔpH sufficiently to trigger an increased resistance to electron transfer through cyt*b*_6_*f* (red lines) and thus oxidation of PSI. This is the canonical mechanism of PCON.

The photosynthetic coupled electron-proton transfer reactions provide the driving force for CO_2_ fixation (Fig. 1A). Linear electron transfer (LET) begins with light-powered water-splitting at PSII with the 2 electrons passed to the mobile acceptor plastoquinone (PQ) and 2 protons taken up from the stroma to form plastoquinol (PQH_2_). PQH_2_ then must diffuse to the cytochrome *b*_6_*f* (cyt*b*_6_*f*) complex, where it is oxidized at the Q_p_ site via the so-called Q-cycle. The first electron is used to reduce the mobile acceptor Pc, and the second is used to reduce another PQ molecule at the reducing (Q_n_) site of cyt*b*_6_*f* (Malone et al. 2021; Sarewicz et al. 2021). A second light-powered reaction at PSI then results in the reduction of ferredoxin (Fd) and oxidation of Pc. Finally, ferredoxin-NADP^+^ (FNR) reductase transfers electrons from Fd to NADP^+^ to form NADPH. Protons are released during water splitting at PSII and PQH_2_ oxidation at cyt*b*_6_*f* to form a transmembrane proton gradient (pmf) (H^+^/e^−^ = 3), which powers the endergonic synthesis of ATP from ADP and Pi by ATP synthase (Fig. 1A). Pmf is composed of the ΔpH (proton concentration difference) and Δψ (charge difference) components, which are thermodynamically equivalent. CO_2_ fixation into carbohydrate in the stroma occurs via the CBB cycle, which consumes ATP to convert 3-phosphoglycerate to 1,3 bisphosphoglycerate (BPG) and ribulose 5-phosphate to to ribulose 1,5- bisphosphate. NADPH is also required by the CBB cycle to convert BPG to triose phosphate (TP). These 3 energy-consuming reactions require 1.5 ATP per NADPH; however, LET produces these in the ratio of 1.28 (6H^+^ moved across thylakoid per NADP^+^ reduced/4.67 H^+^ required per ATP by ATP synthase). Therefore, ATP production must be augmented by another means (Allen 2002; Kramer and Evans 2010). These demands are further altered by the extent of photorespiration, nitrogen and sulfur fixation, and other biosynthetic reactions occurring in the chloroplast (Noctor and Foyer 1998).

Several alternative electron transfer (AET) pathways exist in angiosperms (Table 1) that can contribute to balancing the ATP/NADPH ratio, including cyclic electron transfer (CET), pseudo cyclic electron transfer (PCET), and respiratory electron transfer in the mitochondria (RET) (Allen 2003; Yamori and Shikanai 2015; Chadee et al. 2021). PCET can be catalyzed via several routes, and electrons can be routed from water at PSII back to oxygen via either the Plastid Terminal Oxidase (PTOX) (H^+^/e^−^ = 1), which reduces it to water at the stromal membrane side (Nawrocki et al. 2014; Messant et al. 2024), or PSI can directly reduce O_2_ to superoxide, which is then converted via superoxide dismutase to H_2_O_2_ and to water by ascorbate peroxidase, the so-called Mehler reaction (H^+^/e^−^ = 3) (Miyake 2010). These water-water cycles are the major PCET pathways in angiosperms since they lack the flavodiiron (Flv) proteins found in gymnosperms, moss, and algae that catalyze NADPH to O_2_ electron transfer to produce water (Yamamoto et al. 2016; Chaux et al. 2017; Storti et al. 2020). Alternatively, excess reductant may be exported from the chloroplast via the malate valve (Selinski and Scheibe 2019). Here the chloroplast NADPH-dependent malate dehydrogenase converts oxaloacetate to malate. The malate is then converted in the reverse reaction in mitochondria to produce NADH as a substrate for RET using either Complex IV and O_2_ as a terminal acceptor (H^+^/e^−^ = 5) or alternative oxidase and O_2_ (H^+^/e^−^ = 2) (Lim et al. 2020; Chadee et al. 2021). Under various environmental circumstances, each of these electron fluxes can contribute to balancing the chloroplast ATP/NADPH ratio. However, it is clear that in angiosperms CET carries the major burden of alternative electron flow, as evidenced by the comparative severity of the phenotypes of Arabidopsis mutants lacking various AETs (Munekage et al. 2004; Hebbelmann et al. 2012; Kobayashi et al. 2024). Two major mechanisms of CET exist in angiosperms (Fig. 1A, Table 1), the Proton Gradient Regulation 5 (PGR5)-dependent and NADH-like dehydrogenase complex (NDH-1)-dependent pathways, both of which recycle electrons from Fd to PQ, thus creating a cycle around cyt*b*_6_*f* and PSI (Yamori and Shikanai 2015). The PGR5 pathway has an H^+^/e^−^ ratio of 2 and was first suggested to utilize the PGRL1 protein in complex with PGR5 as a non–proton-pumping Fd-PQ reductase (FQR) (Munekage et al. 2004; DalCorso et al. 2008; Hertle et al. 2013), though recently this idea was dismissed (Rühle et al. 2021). Alternatively, PGR5-dependent CET may involve direct electron donation from Fd or FNR to cyt*b*_6_*f*, with FQR activity occurring at the Q_n_ site and PGR5 acting as a regulator (Joliot and Johnson 2011; Buchert et al. 2020). In contrast, the NDH pathway involves the proton-pumping NDH-1 complex, which is analogous to complex I in mitochondria and raises the H^+^/e^−^ ratio to 4 (Strand et al. 2017) (Fig. 1A).

**Table 1.** AET pathways in angiosperms

AET pathway		Mechanism	H^+^/e^−^	Environmental condition
Pseudo cyclic electron transfer	Plastid Terminal Oxidase	2 PQH_2_ + O_2_ → 2 PQ + 2 H_2_O	1	Salt stress (Stepien and Johnson 2008), light and cold stress (STREB et al. 2005; Ivanov et al. 2012), drought and high temperature (Ibáñez et al. 2009), high UV (Laureau et al. 2012)
	Mehler reaction (Water-Water-Cycle)	O_2_ → O^−^_2_→ H_2_O_2_ + H_2_O	3	High light and/or low CO_2_ (Makino et al. 2002; Miyake 2010)
Malate valve and respiratory electron transfer in the mitochondria		Oxaloacetate + NADPH ↔ malate + NADP+	2–5	Low CO_2_, high and/or fluctuating light (Scheibe 2004; Selinski and Scheibe 2019; Chadee et al. 2021)
Cyclic electron transfer	Proton Gradient Regulation 5 (PGR5)-dependent	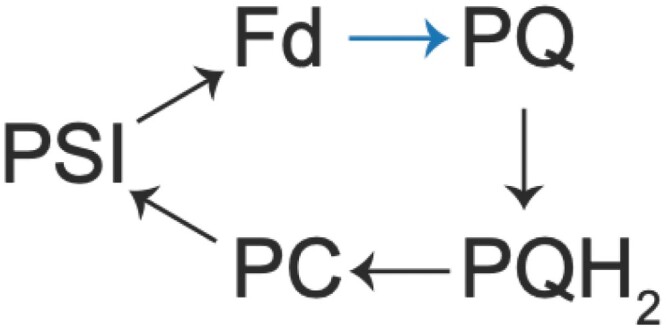	2	Fluctuating and/or high light, high temperature (Nishikawa et al. 2012; Suorsa et al. 2012; Penzler et al. 2022)
	NADH-like dehydrogenase complex 1 (NDH-1)-dependent		4	Low light, low temperature (Yamori et al. 2011, 2015)

The CBB cycle requires 1.5 ATP per NADPH; however, LET produces these in the ratio of 1.28. The AET pathways in angiosperms, the mechanisms, and environmental conditions in which they play a dominant role are summarized. The blue arrow indicates the step where Fd transfers electrons to PQ, which occurs in NDH-1 complex (NDH pathway) or possibly at the cyt*b*_6_*f* complex (PGR5 pathway). For both CET pathways PQH_2_ is oxidized at the cyt*b*_6_*f* complex.

While in low-light conditions, photosynthesis is limited by the production of NADPH and ATP; in excess light, the limitation switches to the CBB cycle reactions and the availability of CO_2_ (Kramer and Evans 2010; Foyer et al. 2012). In these circumstances, NADPH will accumulate and the electron transfer chain will become overreduced, leading to a shortage of electron acceptors at PSII and PSI (Fig. 1B). This can prolong the lifetime of chlorophyll singlet excited states in PSI and PSII, leading to intersystem crossing to the triplet state and reaction with O_2_ to form singlet O_2_ (Krieger-Liszkay 2005). Alternatively, PSI can directly reduce O_2_ to superoxide (**^·^**O_2_^−^) (Sonoike et al. 1995; Asada 1999; Sonoike 2010). On one hand, these ROS act as important secondary messengers informing plant acclimatory responses via changes in gene expression. However, they also have the potential to damage the sensitive photosynthetic machinery of the reaction centers and cause photoinhibition (Li et al. 2009; Foyer and Noctor 2013). Plants must therefore carefully allow sufficient ROS for signaling while avoiding oxidative damage. Defenses against excess ROS generation in the chloroplast include both prevention via the regulation of photosynthesis and cure via antioxidant scavenging pathways (e.g. superoxide dismutase, ascorbate peroxidase, and glutathione peroxidase) (Foyer and Noctor 2011; Foyer and Hanke 2022). In PSII any photo-oxidative damage is somewhat mitigated by the presence of a dedicated repair cycle that allows the damaged RC D1 subunit to be excised, proteolytically digested, and replaced by a fresh protein on a timescale of minutes to hours (Aro et al. 1993; Theis and Schroda 2016). In addition, in excess light, the ΔpH acts to trigger the dissipation of excess excitation energy in the light-harvesting complexes (LHCII) of PSII via the protonation of the PsbS and violaxanthin de-epoxidase (VDE) proteins, a process known as energy-dependent nonphotochemical quenching (qE) (Holt et al. 2004; Ruban et al. 2012). VDE catalyzes the conversion of the LHCII-bound xanthophyll violaxanthin to zeaxanthin, and together with PsbS, this brings about conformational change in LHCII that triggers qE, protecting RCs from over-excitation (Ruban et al. 2012).

In contrast, however, PSI lacks a dedicated repair cycle, and therefore any photo-induced damage is long-lived since it requires the synthesis and assembly of an entirely new complex on a timescale of days to weeks (Sonoike et al. 1995; Zhang and Scheller 2004; Sonoike 2010; Tiwari et al. 2016). PSI photoinhibition therefore has the potential to strongly impact growth by unbalancing LET transfer and reducing its flux (Suorsa et al. 2012; Zivcak et al. 2015; Lima-Melo et al. 2019; Lempiäinen et al. 2022).

## Photo-oxidative stress in PSI

**Figure 2.**
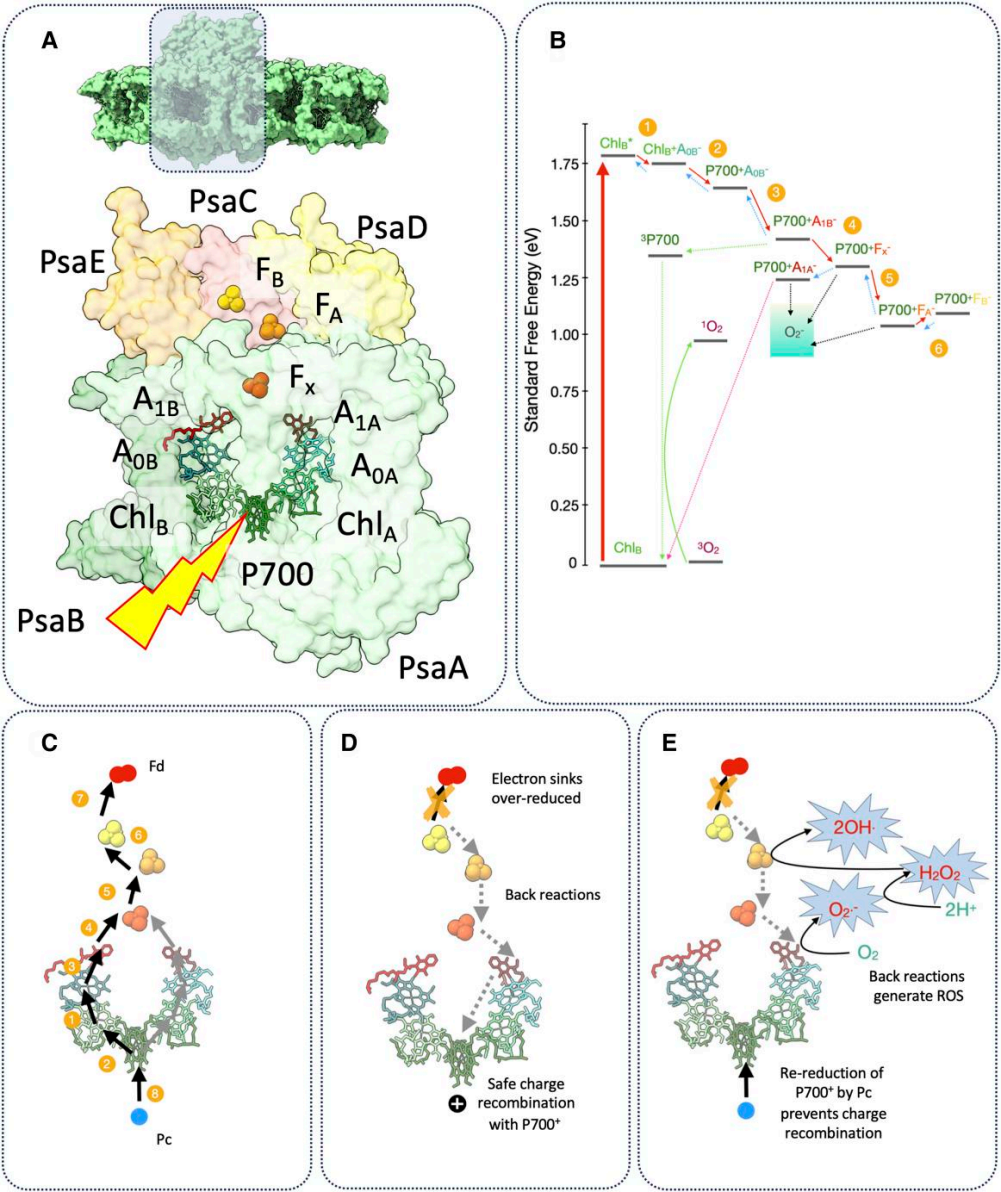
Electron transfer reactions of PSI give rise to reactive oxygen species and photoinhibition. **A)** Organization of the PSI reaction center (RC) core complex subunits PsaA, PsaB, PsaC, PsaD, and PsaE. The core cofactors of the electron transfer chain in PSI are labeled. **B)** Standard free energy changes (eV) for the PSI forward (red arrows) and back (blue arrows) reactions. Crossover to the P700 triplet state and formation of singlet oxygen are shown in green arrows; safe charge recombination between P700^+^ and A_1A_^−^ (pink arrow) and reduction of molecular oxygen to superoxide (black arrows) are also highlighted. The numbered yellow circles refer to the steps shown in **C)**. Forward electron transfer reactions in the PSI RC. The numbers marked refer to the sequence of steps outlined in C and explained further in the text. **D)** When downstream electron sinks Fd, NADPH, etc. are overreduced, the back-reactions within the RC occur with increased frequency. Maintenance of P700 in an oxidized state by PCON allows excess electrons to be safely dissipated as heat via charge recombination. **E)** In the absence of PCON, Pc re-reduces P700^+^ before charge recombination can occur. Reduced acceptors in PSI can react with O_2_ to generate superoxide, hydrogen peroxide, and hydroxyl radicals via the Fenton reaction.

The structure of the PSI reaction center includes the special pair chlorophylls P700, the primary donor chlorophylls Chl_A_ and Chl_B_, primary chlorophyll acceptors A_0A_ and A_0B_, the secondary acceptor phylloquinones A_1A_ and A_1B_, and finally three 4Fe4S iron-sulfur clusters: F_x_, F_A_, and F_B_ (Fig. 2A) (Amunts et al. 2007; Mazor et al. 2015). Light drives excitation (*) of the chlorophylls within PSI, which leads to charge separation generating an electron (−) and hole (+). Electron transfer can in principle occur along either A or B branch of the PSI RC following the scheme Chl* > 1) Chl^+^A_0_^−^ > 2) P700^+^A_0_^−^ > 3) P700^+^A_1_^−^ > 4) P700^+^F_x_^−^ > 5) P700^+^ F_A_^−^ > 6) P700^+^ F_B_^−^> 7) P700^+^ Fd^−^> 8) P700 Pc (Fig. 2, B and C) (Brettel 1997; Brettel and Leibl 2001; Li et al. 2006; Müller et al. 2010). In steps 7 and 8, the F_B_ acceptor reduces the 2Fe2S soluble protein Fd, while P700^+^ is re-reduced by Pc (Fig. 2C). A shortage of electron acceptors due to saturated CO_2_ fixation increases the probability of charge recombination, the back-reaction between the electron and hole that results in loss of energy as heat (Fig. 2, B and D) (Brettel and Leibl 2001). The recombination reaction between A_1B_^−^ and P700^+^ that occurs on a timescale of 200 µs can generate singlet oxygen via the formation of the P700 triplet state. This is largely mitigated via the redox tuning of A_1A_, which has an energy below that required for P700 triplet formation (Rutherford et al. 2012) (Fig. 2B). Thus the back-reaction from F_x_ and F_A_/F_B_ leads to the formation of A_1A_^−^ rather than A_1B_^−^ and hence avoids P700 triplet states, safely recombining with P700^+^ and returning to the ground state (Fig. 2D). Therefore, the production of singlet O_2_ is suppressed. Under circumstances where P700^+^ is first re-reduced by Pc, then the low redox potential of the 4Fe4S clusters and phylloquinones means that they are capable of reducing O_2_ to **^·^**O_2_^−^ (−160 mV) (Sonoike et al. 1995; Asada 1999; Mubarakshina and Ivanov 2010; Sonoike 2010; Kozuleva et al. 2021). Dismutation of **^·^**O_2_^−^ radicals into H_2_O_2_ leads to their conversion by 4Fe4S clusters into **^·^**OH^−^ hydroxyl radicals via the Fenton reaction, subsequently destroying the clusters (Fig. 2E) (Takahashi and Asada 1988; Sonoike et al. 1997). Destruction of the 4Fe4S clusters is the primary cause of damage to the PSI RC, as confirmed by electron paramagnetic resonance spectroscopy (Sonoike et al. 1995; Tiwari et al. 2016; Furutani et al. 2022). There is evidence that damage to F_A_ and F_B_, which lie in the stromal side membrane-associated proteins PsaC, D, and E, can be recovered within ∼24 hours; yet recovery of the damage to F_x_ (bound to the PsaA/B core heterodimer) takes much longer since it requires degradation and synthesis of an entirely new PSI complex (Tiwari et al. 2016; Lempiäinen et al. 2022). Since methyl viologen, which accepts electrons from PSI acceptors and in turn reduces O_2_ to **^·^**O_2_^−^, alleviates PSI photoinhibition, it is clear that the site of ROS production is crucial to the outcome (Takagi et al. 2016).

At a physiological level, photoinhibition of PSI is commonly diagnosed as a decrease in the amount of photo-oxidizable P700 (measured either as photobleaching at 700 nm or absorption via the P700^+^ cation at 820 nm) (Sonoike 2010; Suorsa et al. 2012; Takagi et al. 2016; Tiwari et al. 2016). PSI photoinhibition has been observed in cucumber, cotton, coffee, and common bean subjected to high light and cold stress and was later also seen in Arabidopsis (Sonoike et al. 1995; Ramalho et al. 1999; Kornyeyev et al. 2003; Zhang and Scheller 2004; Nakano et al. 2010). Experiments in vitro on thylakoids and chloroplasts show PSI photoinhibition to be inhibited by 3-(3,4-dichlorophenyl)-1,1-dimethylurea and dibromothymoquinone, which block electron transfer at PSII or at cyt*b*_6_*f*, respectively (Sonoike 1996). Consistent with this, results in vivo show that Arabidopsis mutants suffering PSI photoinhibition can be rescued by decreasing the electron flow from PSII (Suorsa et al. 2016; Penzler et al. 2022). Recently, a new methodology has emerged for studying PSI photoinhibition that utilizes repetitive short pulse illumination (rSP) (Sejima et al. 2014; Tikkanen and Grebe 2018). Since the pulses are not long enough to generate significant ΔpH, they can cause overreduction of PSI acceptors and photodamage. Consistent with the involvement of ROS, PSI photoinhibition is completely suppressed under rSP conditions by lowering O_2_ partial pressure from 21kPa to 2kPa (Sejima et al. 2014; Tikkanen and Grebe 2018; Furutani et al. 2022). Interestingly, under rSP conditions, PSI was able to generate singlet O_2_ in addition to superoxide in line with the method's ability to overwhelm the natural defenses (Takagi et al. 2016).

## Canonical mechanism of PCON

**Figure 3.**
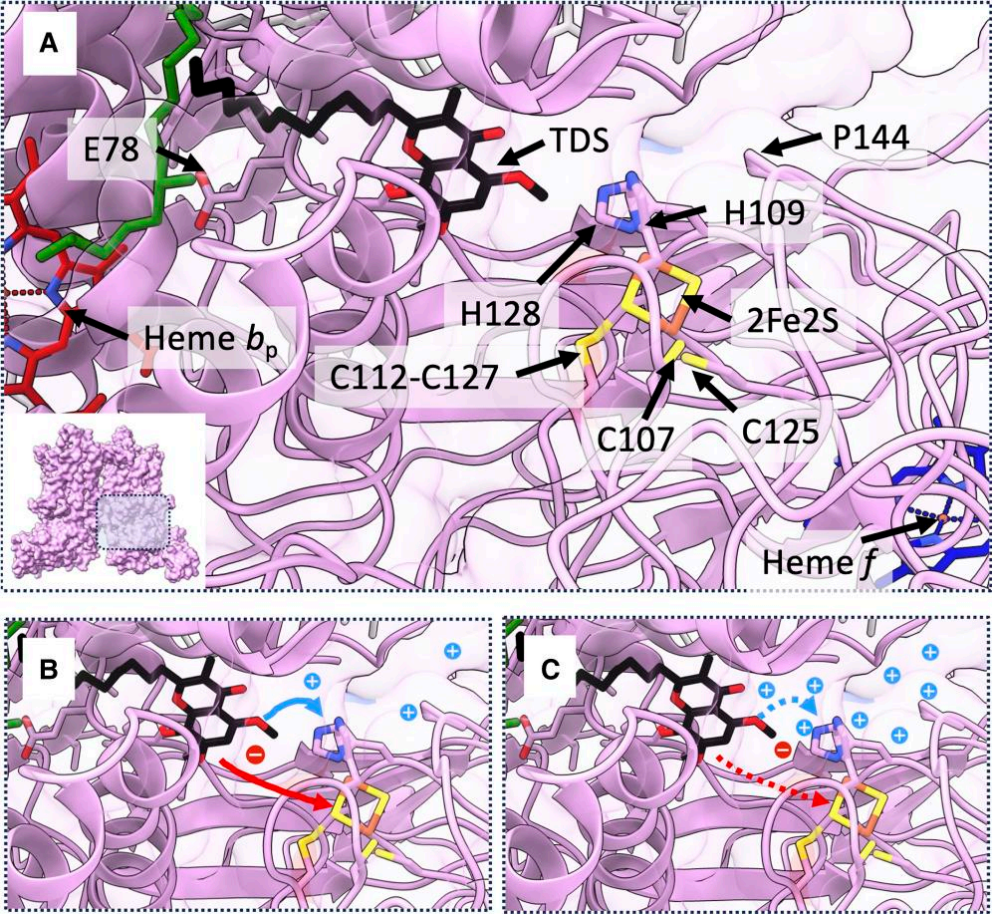
Mechanism of photosynthetic control at the cyt*b*_6_*f* complex. **A)** Zoomed view of the Q_p_ site (PQH_2_ oxidizing site) of cyt*b*_6_*f*. The positions of hemes *b*_p_ and *f*, the second proton accepting residue E78 of subunit IV of cyt*b*_6_*f* and the 2Fe2S ISP ligands H109, H128, C107, and C125 are also shown. The position of PQH_2_ is inferred by the binding of the inhibitor tridecylstigmatellin (TDS), shown in black (Hasan et al. 2013). The possible role of the disulphide bridge between C112 and C127 is discussed in the text. **B)** The pKa of the His ligand (H128) of the 2Fe2S ISP cluster is ∼6.2 when oxidized and ∼8.0 when reduced; therefore, under conditions where the lumen pH > 6.2 the His ligand is unprotonated and therefore able to deprotonate PQH_2_ facilitating cyt*b*_6_*f* turnover. Protons shown in blue, the electron in red. **C)** In excess-light conditions where lumen pH < 6.2, the His128 ligand is protonated irrespective of whether 2Fe2S is oxidized or reduced. This disrupts proton abstraction and thus oxidation of PQH_2_ slowing onward electron transfer toward PSI and thus generating P700^+^. In the Arabidopsis P194L (ISP) mutant (P144 in spinach as shown in panel **A**) without the 50-residue chloroplast targeting sequence included in Arabidopsis numbering), the pKa of the His ligands of the 2Fe2S ISP cluster is upshifted by 1 pH unit, resulting in increased PCON (even under low light).

The primary protection against PSI photoinhibition is that provided by PCON. PCON can be measured in leaves and chloroplasts as a change in the P700 reduction half-time (P700_red_ t½) or its reciprocal, the rate constant (K_P700_). As K_P700_ decreases, so the fraction of PSI in the oxidized state (P700^+^ A)—often referred to as Y(ND) since it is limited by electron donation to the donor side—increases, that is, the P700 oxidation rate exceeds the reduction rate. The remaining fractions of PSI will be split between those that are limited by the acceptor side (P700 A^−^), referred to as Y(NA), and those that are limited by neither side (P700 A), referred to as Y(I), and thus can be oxidized with application of a saturating flash (Klughammer and Schreiber 1994). In a normal healthy leaf from Arabidopsis, one generally observes a gradual decline in Y(I) with light intensity and an increase in Y(ND); meanwhile, Y(NA) generally peaks at low light and declines to a steady level in high light (Barbato et al. 2020; Hepworth et al. 2021). The explanation for the tendency for PSI to become more oxidized with increasing light intensity lies in the slowest step of LET lying between the 2 photosystems at the cyt*b*_6_*f* complex (Stiehl and Witt 1969; Haehnel 1976). Maintaining P700 in an oxidized state allows the safe charge recombination with A_1A_^−^ (Rutherford et al. 2012), thus delaying the re-reduction of PSI by Pc serves a photoprotective function when onward electron acceptors are saturated with electrons. The rate-limiting step of LET involves the oxidation of PQH_2_ by cyt*b*_6_*f* at the Q_p_ site and reduction of Pc, a reaction that involves the deposition of protons into the thylakoid lumen and is thus subject to the thermodynamic back pressure from the pmf (Rumberg and Siggel 1969; Stiehl and Witt 1969). Passage of the electron from the Q_p_ site to Pc is via 2 cofactors of cyt*b*_6_*f*, the 2Fe2S cluster of the Rieske Iron-sulphur protein (ISP) subunit, and the *c*-type heme *f* of cytochrome *f* (cyt *f*) (Fig. 3A). The primary proton-accepting group at the Q_p_ site is believed to be the H128 (spinach numbering) of ISP, which coordinates the 2Fe2S cluster (Fig. 3A). The pKa for this side-chain varies with the redox state of the 2Fe2S cluster from ∼6.2 when oxidized to ∼8 when reduced (Soriano et al. 2002; Tikhonov 2014; Malone et al. 2021). The redox potential difference for the PQH_2_ to cyt *f* reaction is ∼300 mV and since the H^+^/e^−^ = 2 for this complex, the thermodynamic driving force is theoretically maxed out at ∼150 mV pmf; thus, one would expect the reaction to be quite sensitive to small changes in the lumenal pH in addition to the PQH_2_ concentration (Hope et al. 1994; Finazzi 2002). However, if proton exit from cyt*b*_6_*f* is contingent on Pc reduction, then the driving force is increased to 380 mV/2 e^−^.

Rumberg and Siggel (Rumberg and Siggel 1969) were the first to observe that the rate of LET (to the artificial acceptor ferricyanide) was affected by the magnitude of ΔpH in isolated thylakoids. They observed that the P700_red_ t½ was increased and the LET rate decreased as ΔpH increased (Fig. 3, B and C). This effect was abolished by an uncoupler and diminished by active synthesis of ATP, both of which increased the proton efflux. Notably, most of the increase of P700_red_ t½ was found below a predicted lumen pH of 6.0 (Rumberg and Siggel 1969; Siggel 1976; Kobayashi et al. 1979; Tikhonov et al. 1981), consistent with the pKa of the oxidized 2Fe2S ISP cluster. Similar data were later obtained using measurement of cyt *f* reduction half-time (cyt *f*_red_ t½) in intact chloroplasts (Nishio and Whitmarsh 1993).

Further support for the canonical model of PCON was obtained from the Arabidopsis *pgr1* mutant, which has a P194L mutation in the ISP subunit (Fig. 3A) (Jahns et al. 2002). This was suggested to shift in the pKa of the His128 2Fe2S ligand by 1 pH unit and thus allowed activation of PCON at a lower ΔpH than observed in the WT. Indeed, the *pgr1* mutant shows a high steady-state P700 oxidation phenotype, with a restriction on LET due to over-engaged PCON (Yamamoto and Shikanai 2018).

## Relative importance of proton influx and efflux reactions to regulation of PCON

In principle, ΔpH can be raised either by increasing proton influx via electron transfer into the lumen or decreasing the proton efflux via regulation of ATP synthase (Fig. 1B). As noted above, PGR5-dependent CET is the major route for the augmentation of ΔpH in angiosperms (Wang et al. 2015, 2018; Nakano et al. 2019). In line with this, the Arabidopsis *pgr5* mutant possesses a low ΔpH phenotype and shows a complete absence of steady-state P700 oxidation in high light (Munekage et al. 2004; Yamamoto and Shikanai 2018). Consequently, *pgr5* suffers from PSI photoinhibition in excess light (Suorsa et al. 2012). The detrimental effect of *pgr5* mutation on PCON suggested that proton influx catalyzed by PGR5-dependent CET may be crucial. However, the *pgr5* mutant also showed an increase in ATP synthase conductivity (gH^+^), that is, the rate of proton efflux from the lumen, compared with the WT (Avenson et al. 2005). Correspondingly, enhanced ΔpH and restricted gH^+^ in tobacco mutants expressing an antisense construct to *atpc*1, the γ-subunit of ATP synthase, caused an increased steady-state P700 oxidation and increased P700_red_ t½ (Rott et al. 2011). Arabidopsis plants lacking the CGL160 protein, which supports the assembly of ATP synthase and shows lower gH^+^ and higher pmf, also had enhanced steady-state P700 oxidation (Fristedt et al. 2015; Galvis et al. 2020). These results suggested a key role for the control of proton efflux in PCON regulation and a possible additional function of PGR5 in directly regulating gH^+^ at the ATP synthase complex.

Recently, the relative importance of efflux vs influx and the possible role of PGR5 in gH^+^ regulation have been addressed. The *hope2* mutant of Arabidopsis, which carries a mutation in the ATP synthase γ-subunit, suffers from constitutively high gH ^+^ . Interestingly, despite higher proton efflux, *hope2* maintained ΔpH at WT values via an increase in PGR5-dependent CET, yet PCON remained negligible (Degen et al. 2023a, 2023b). Therefore, prima facie, high PGR5-dependent CET activity cannot induce PCON if gH^+^ remains high. Similarly, overexpression of the Chlamydomonas (*Chlamydomonas rheinhardtii*) PTOX in a *pgr5* background was unable to restore steady-state P700 oxidation despite restoration of ΔpH, while gH^+^ remained high (Zhou et al. 2021). An essential role for PGR5 in regulating gH^+^ or PCON directly could also be ruled out since overexpression of *Physcomitrium patens* FLVA and B proteins, which transfer electrons from NADPH to O_2_, in the *pgr5* background restored steady-state P700 oxidation, gH^+^, and pmf (Yamamoto et al. 2016). Similarly, the *pgr1 pgr5* double mutant showed a WT-like gH^+^, though it lacked steady-state P700 oxidation due to a lower ΔpH (Yamamoto and Shikanai 2020). Equally, infiltration of *pgr5* leaves with methyl viologen also restored these parameters to WT values (Munekage et al. 2002; Wang et al. 2018). Therefore, seemingly only when WT gH^+^ regulation and WT-like or enhanced ΔpH are combined is steady-state P700 oxidation in excess light observed.

## Distinguishing PCON from P700 oxidation [Y(ND)]

The examples given above from the literature suggest that the regulation of PCON may be rather more complex than suggested by the canonical model. Moreover, measurements of P700^+^ absorption at 820 nm on leaves showed that contrary to the in vitro situation, the P700_red_ t½ and K_P700_ were invariant with irradiance in pea (Harbinson and Hedley 1989). Interestingly, over the same range of light intensity, the steady-state P700 oxidation varied from 0% to 80%, suggesting that changes in Y(ND) are not exclusively reliant on changing the rate of the PQH_2_ oxidation reaction at cyt*b*_6_*f*. Rather, it suggests a constant cyt*b*_6_*f* resistance with oxidation of P700 simply driven by the increasing light intensity, that is, oxidation of P700 and onward transfer to PSI acceptors exceeds electron donation via cyt*b*_6_*f*. Consistent with this, Kramer et al. (1999) also found an invariant cyt *f*_red_ t½ in leaves with changing light intensity. Numerous other examples exist in the literature that also contradict the simple canonical model for PCON regulation. For instance, a wide range of qE values, inferring changing ΔpH, can be observed without any change in the K_P700_. On the other hand, changes in K_P700_ are sometimes observed without any corresponding increase in qE (Ott et al. 1999; Johnson 2003; Hald et al. 2008).

**Figure 4.**
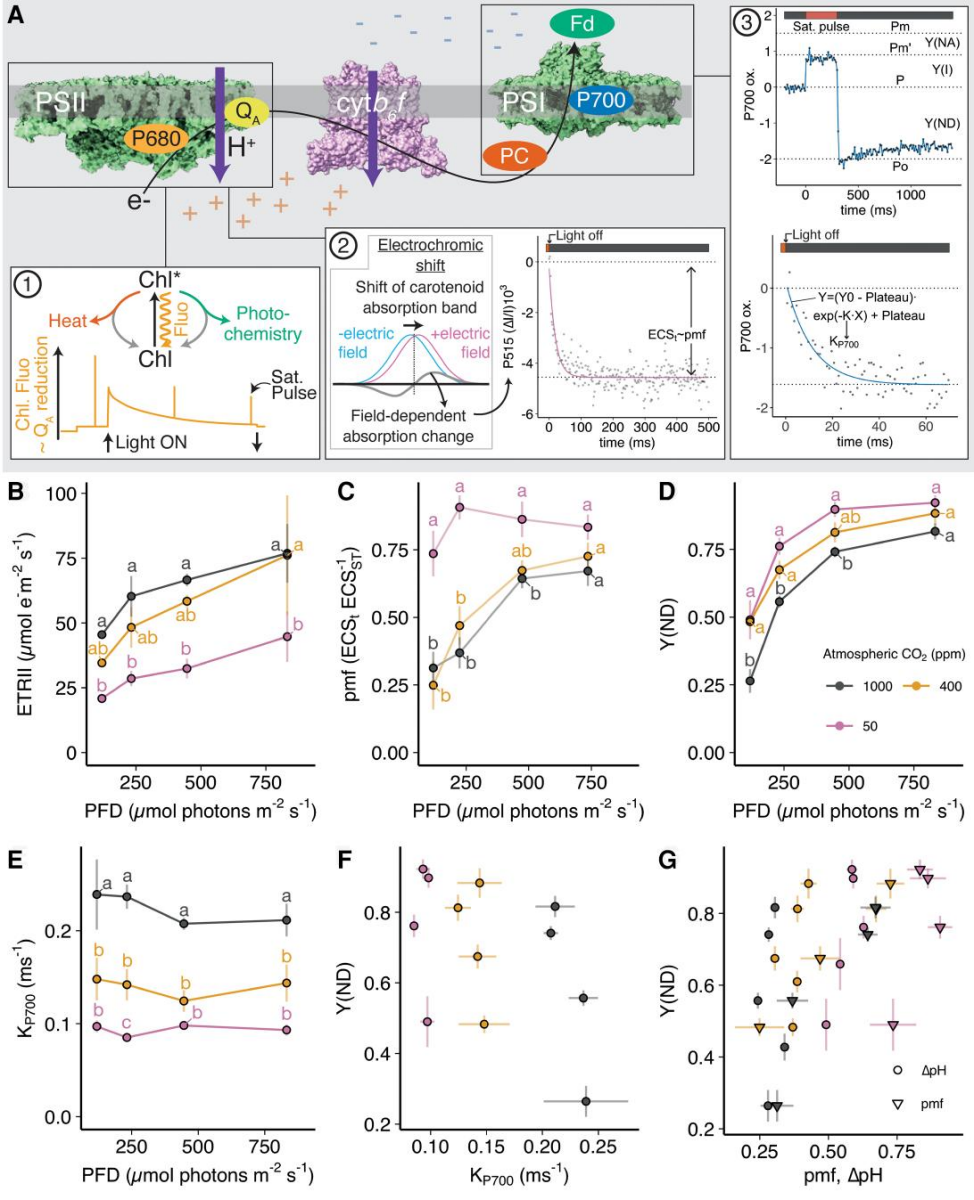
Overview of measurement techniques and photosynthetic parameters of Arabidopsis at 3 CO_2_ concentrations. **A)** 1: The excited Chlorophyll (Chl*) can return to its ground state via the photochemical route, the nonphotochemical route, or emitted as fluorescence. Chlorophyll fluorescence is measured using a PAM-fluorometer and the activity of PSII is assessed by application of saturating pulses. The illustration shows a fluorescence trace during exposure to actinic light. An increase in fluorescence indicates greater reduction of the PSII primary acceptor Q_A_, that is, less energy is used in photochemistry and is instead emitted as fluorescence. 2: Upon illumination, the absorption band of carotenoids in the photosynthetic complexes undergo a shift, referred to as ECS. The field-dependent absorption change in Arabidopsis is measured at 515 nm and provides a measure of the proton motive force across the thylakoid membrane. This is quantified by applying short dark pulses (<1 s) to illuminated leaves and fitting an exponential decay. The span of the fit is termed ECS_t_ and proportional to pmf, once normalized to a single turnover flash. The rate constant K is proportional to the proton conductance (gH^+^) of the thylakoid membrane. 3: The saturation pulse method is used to determine PSI quantum yield and DIRK method for P700 re-reduction. Maximal P700 oxidation (Pm, equivalent to maximal fluorescence (Fm) in PSII) is determined before the measurement by a combination of far-red light and a high-intensity flash. During light curves, saturation pulses allow for partitioning into Y(I) (fraction of open reaction centers), Y(ND) (fraction of closed reaction centers due to donor-side limitation), and Y(NA) (fraction of closed reaction centers due to acceptor-side limitation). P700_red_ t½ was calculated by fitting an exponential decay function to the data points, where K is the rate constant of the decay. In B to G the data were collected on Arabidopsis wild-type (Col-0) leaves at 3 different CO_2_ concentrations (50, 400, and 1000 ppm CO_2_), and points represent the mean of 3 to 5 biological replicates ±SEM at increasing light intensities. Means were compared between CO_2_ conditions at each light intensity using an ordinary 2-way ANOVA and corrected using Tukey multiple comparison test followed by an HSD test with alpha = 0.05. Different letters indicate significant differences between data points. **B)** Electron transport rate through PSII (ETRII), **C)** Proton motive force (pmf) calculated from the decay of the electrochromic shift signal during a brief dark period, normalized to the height of a 50-µs single turnover flash applied before measurements. **D)** PSI oxidation or donor side-limitation (YND). **E)** Rate constant (K) of P700 reduction following an 80-ms dark pulse under the same conditions. **F)** Relationship between YND and K_P700_. **G)** Relationship between K_P700_ and pmf or nominal ΔpH (see text).

These examples argue against a simple model where ΔpH alone regulates PCON via changing the resistance at the cyt*b*_6_*f* complex. In Fig. 4A we highlight the key photosynthetic signals (electrochromic shift [ECS], chlorophyll fluorescence, and P700 absorption) that can be obtained from intact leaves to investigate PCON. Chlorophyll fluorescence-derived PSII electron transfer rate (Fig. 4B) and pmf derived from ECS measurements (Fig. 4C) demonstrate the increase in these parameters with light intensity at atmospheric CO_2_ concentrations of 400 ppm. Similarly, Y(ND), the fraction of P700 oxidized in the steady state, also increases across this range (Fig. 4D). However, at the same CO_2_ concentration, K_P700_ is invariant with light intensity consistent with the pattern previously observed by Harbinson and Hedley (1989). Therefore, there is a clear need to disentangle references to PCON and Y(ND) in the literature. Evidence of PCON per se requires measurement of the K_P700_. Without this measurement, there can be no automatic assumption that increased steady-state P700 oxidation [Y(ND)] reflects a change in the resistance of the cyt*b*_6_*f* complex.

Further clues to unraveling the regulation of PCON might be gleaned from those conditions, which *do* bring about altered resistance of the cyt*b*_6_*f* complex and a change in K_P700_. Alterations in K_P700_ were observed during photosynthetic induction and with varying CO_2_ concentration and temperature (Harbinson and Hedley 1989; Harbinson and Hedley 1993; Ott et al. 1999; Hald et al. 2008). The K_P700_ increased with increasing CO_2_ and decreased during photosynthetic induction, stabilizing after ∼90 s. The effect of temperature was more subtle, though variance in K_P700_ was generally observed with varying light intensity at higher temperatures >20°C. We observe a similar variance of K_P700_ with changing CO_2_ concentration (Fig. 4E), paralleled by changes in Y(ND), pmf, and ETRII (Fig. 4, C and D). At 50 ppm, K_P700_ is decreased by approximately one-third and correspondingly steady-state P700 oxidation is higher, whereas the opposite is true at 1000 ppm, where K_P700_ is increased by ∼60% while P700 oxidation is suppressed (Fig. 4, D and E). Crucially, no correlation exists between Y(ND) and K_P700_ (Fig. 4F). Similarly, no correlation is seen between pmf and Y(ND) either (Fig. 4G). The exact partitioning of pmf in plant chloroplasts between ΔpH and Δψ remains under debate, with recent evidence showing the ECS signal is corrupted by contributions from qE- and zeaxanthin-related absorption changes, which make disentangling the signals difficult (Johnson and Ruban 2014; Wilson et al. 2021). Indeed, when these contributions were removed, pmf was almost entirely partitioned into ΔpH (Wilson et al. 2021), consistent with experiments on intact chloroplasts (Vredenberg 1997). Nonetheless, even when the ECS signal is partitioned into nominal ΔpH as per Kramer et al. (2003), still no correlation is observed with Y(ND) in leaves (Fig. 4G). Collectively, these data show that no simple relationship exists in intact leaves between K_P700_, Y(ND) and pmf. Importantly, a change in the metabolic state of the chloroplast stroma brought about by altered CO_2_ concentration was able to modulate PCON.

## A modified PCON mechanism based on dual redox and ΔpH control

Several examples in the literature argue for a more complex regulation, which takes into account both ΔpH and the redox/metabolic state of the chloroplast stroma. Firstly, antisense glyceraldehyde-3-phosphate dehydrogenase (GAPDH) tobacco mutants were limited in photosynthesis due to lower CBB cycle activity and responded by inducing qE and PCON, whereas antisense FNR tobacco mutants, which also possess decreased CO_2_ fixation, showed normal levels of qE and, by inference ΔpH though were unable to induce PCON (Hald et al. 2008). A key difference was that the NADP^+^/NADPH pool was more oxidized in the FNR antisense but showed WT levels of reduction in the GAPDH antisense. This suggests a key role for NADP^+^/NADPH redox poise in regulating PCON. Indeed, (Johnson 2003) demonstrated that the ΔpH sensitivity of PCON in vitro was sensitive to redox poise, with a regulatory element with a midpoint potential of −365 mV (i.e. between that of NADPH and Fd).

Recently, an alternative approach to the problem of PCON was suggested based on measuring the electron flux into and out of the PQ pool using PSII fluorescence as a proxy (Johnson and Berry 2021). As the light intensity increases, more electrons are driven into the PQ pool by PSII and 1-qL (the redox state of the PSII acceptor Q_A_ and by inference the PQ pool) increases. Using short 300-ms flashes that provoke little change in NPQ and, by inference ΔpH, they showed that the apparent conductivity of cyt*b*_6_*f* i.e. LET/1-qL is rapidly decreased with increasing PQ pool reduction. Other supporting observations have been made in cyanobacteria, where the oxidation of PSI was alleviated by the addition of H_2_O_2_ as an electron acceptor to ascorbate and glutathione peroxidases, which can thereby re-oxidize NADPH and thus the PQ pool (Shimakawa et al. 2018). In principle, since these peroxidase reactions do not consume ATP, they should not diminish ΔpH, suggesting re-oxidation of the PQ pool alone can relax PCON. Consistent with this, H_2_O_2_ increased ΔpH-dependent qE in spinach chloroplasts (Backhausen et al. 2000). The authors proposed a mechanism of redox regulation based on the inhibition of the Q_n_ site of cyt*b*_6_*f*, which catalyzes PQ reduction during the Q-cycle. If the PQ concentration is too low then the Q-cycle and so onward electron transfer to P700 may be inhibited, the so-called reduction-induced suppression of electron flow (Shimakawa et al. 2018).

In the model green alga Chlamydomonas, the balance between Y(ND) and Y(NA) is generally more skewed to the latter compared with plants under permissive oxic conditions (Ozawa et al. 2022). Indeed, the higher PCON phenotype of the equivalent *pgr1* mutant in Chlamydomonas is only observed under anoxic conditions (Ozawa et al. 2022). This difference in regulation between oxic and anoxic conditions may reflect an increased partitioning of pmf toward ΔpH under anoxia, thus inducing PCON (Finazzi and Rappaport 1998). In the Chlamydomonas *pgr5* mutant, a higher K_P700_ is observed under anoxia compared with the wild-type, indicative of perturbed PCON (Buchert et al. 2020, 2022). Interestingly, the study pointed to a role for PGR5 in redox-dependent regulation of the Q-cycle of cyt*b*_6_*f* (Buchert et al. 2020, 2022). Similarly, a ΔpH-independent block on electron flow through cyt*b*_6_*f* has been observed in Chlamydomonas mutants lacking starch synthesis placed under nitrogen stress, again suggesting an additional redox-based regulation of PCON (Saroussi et al. 2023).

A mechanistic basis for redox regulation of PCON was proposed by Hald et al. (2008). They suggested the ISP C112-C127 disulfide bridge in cyt*b*_6_*f* (Fig. 3A) could act to regulate the pKa of the 2Fe2S cluster ligand H128 and thus the de-protonation of PQH_2_ at the Q_p_ site. The position of these cysteine residues is conserved in all ISP genes, including those of the similar cytochrome *bc*_1_ complex and is known to have a low potential and interact with thioredoxin proteins in vitro (Merbitz-Zahradnik et al. 2003; Zu et al. 2003; Buchanan and Luan 2005; Leggate and Hirst 2005; Balmer et al. 2006). When reduced, the C112 and C127 could provide 2 additional ligands to the 2Fe2S cluster causing a conformational change, simultaneously stabilizing the oxidized cluster and raising the pKa of the His128 ligand, allowing its protonation at higher lumenal pH. Reducing power can be delivered to the lumen from the stroma via the thiol/disulfide membrane transporter CcdA and HCF164 a membrane-anchored, lumen-facing, thioredoxin-like protein (Page et al. 2004). Re-oxidation may in turn occur via the Lumen Thiol Oxidoreductase1 (LTO1) (Karamoko et al. 2011). Such a mechanism would link the redox state of the stroma with PCON, ensuring its rapid activation upon sudden shifts in light as observed by Johnson and Berry (2021). A dual requirement of stromal redox poise and ΔpH for PCON activation would be elegant, as it would ensure that maximum rates of ATP synthesis and electron transfer can coexist in permissive conditions while allowing rapid downregulation under environmental stress.

## Does the transfer of electrons from cyt*b*_6_*f* to P700 via Pc limit electron transfer under certain conditions?

While the oxidation of PQH_2_ at the Q_p_ site of cyt*b*_6_*f* is the slowest step in LET, there is evidence that under certain conditions Pc diffusion to and unbinding from PSI can also limit photosynthesis (Kirchhoff et al. 2011; Höhner et al. 2020; Hepworth et al. 2021). Lateral heterogeneity exists in the organization of thylakoid components with PSII largely confined to stacked grana regions of the membrane, while PSI and ATP synthase are confined to the unstacked interconnecting stromal lamellae membranes (Daum et al. 2010; Austin and Staehelin 2011; Wietrzynski et al. 2020). Cyt*b*_6_*f* in contrast resides in both regions of the membrane in roughly equal proportions, and therefore the grana and stromal lamellae fractions of the complex are spatially separated from their PSI electron acceptor or PSII electron donor respectively. The diffusion distances for the mobile electron carriers PQ/PQH_2_ and Pc can therefore be upwards of 300 to 600 nm between these 2 membrane domains. Since both the thylakoid membrane itself and the lumen space are highly protein crowded, this creates the potential for diffusion limitations on LET (Kirchhoff 2014; Garty et al. 2024). Interestingly, mutants of Arabidopsis showed a positive correlation between grana diameter and P700_red_ t½ upon either single flash excitation or under steady-state light conditions (Hepworth et al. 2021). Since these structural changes occurred without substantial differences in ΔpH, it suggested that at least for the mutants above a certain threshold LET is influenced by diffusion limitations. Indeed, disequilibrium has been recorded between the redox states of cyt *f*/Pc and P700 in vivo, with increasing grana size increasing its severity (Joliot and Joliot 1984; Kirchhoff et al. 2004; Golding et al. 2005; Höhner et al. 2020; Hepworth et al. 2021). Since grana size is reversibly regulated by STN7 kinase-dependent phosphorylation of LHCII proteins, dynamic thylakoid stacking provides another ΔpH-independent means of regulating P700 oxidation and reduction (Wood et al. 2018, 2019; Hepworth et al. 2021; Garty et al. 2024). Indeed, STN7 provides a further link between the stromal redox state and cyt*b*_6_*f* lumenal ISP domain and there is evidence that it is regulated in a redox-dependent manner via a disulfide bridge between the C65 and C70 residues (Lemeille et al. 2009; Shapiguzov et al. 2015; Dumas et al. 2017). In addition to distance-dependent diffusion limitations, it was shown in Chlamydomonas that unbinding of oxidized Pc from PSI is also pH regulated and can limit LET in mutants with modified binding interfaces between these 2 proteins (Finazzi et al. 2005; Kuhlgert et al. 2012).

## Potential for future manipulation of P700 oxidation to improve crops

In contrast to ΔpH-dependent qE, whose dynamics show hysteresis with respect to changes in light intensity, P700 oxidation relaxes extremely rapidly (Ruban et al. 2012; Shimakawa and Miyake 2018). Indeed, using a rapidly oscillating sine wave type illumination regime on Arabidopsis plants demonstrated that while qE was largely unable to rapidly and accurately track changes in light intensity, PCON was much more responsive (Shimakawa and Miyake 2018). A priori, this suggests that transgenic approaches employed to successfully increase crop yield by manipulating the rate of qE relaxation (Kromdijk et al. 2016) to better track changing light intensity are unnecessary for P700 oxidation. However, overexpression of the ISP subunit and thus increased levels of the cyt*b*_6_*f* complex in *Setaria viridis* and Arabidopsis were shown to increase growth and CO_2_ assimilation under high-light conditions, suggesting increasing electron flow to PSI can enhance photosynthesis (Simkin et al. 2017; Ermakova et al. 2019). Indeed, the Setaria ISP overexpressors showed higher PSI yield and lower PSI oxidation yet higher growth compared with the WT. Similarly, the expression of algal cytochrome c_6_, an alternative electron carrier to Pc between cyt*b*_6_*f* and PSI, could also increase growth (Chida et al. 2007). It is therefore likely that re-tuning the sensitivity of cyt*b*_6_*f* to ΔpH, perhaps making this system less resistant to electron flow, could work in synergy with manipulation of downstream electron sinks, for example, via overexpression of rate-limiting enzymes in the CBB cycle to improve crops (López-Calcagno et al. 2020).

## Conclusions

The importance of P700 oxidation [Y(ND)] for avoidance of PSI photoinhibition is clear. However, PCON in the classical sense—a change in resistance of cyt*b*_6_*f* to electron flow in response to ΔpH alone—is not always responsible since K_P700_ or P700_red_ t½ is often unchanged with increasing light intensity. Rather, resistance is constant, and therefore Y(ND) reflects the inherent, though unchanged, limitation cyt*b*_6_*f* places on the rate of LET. This highlights the distinct difference between PCON and Y(ND). Hence to assign a change in PCON, PSI re-reduction rates are required (P700_red_ t½) and even more precisely to implicate a change in resistance of cyt*b*_6_*f* measurement of cyt *f*_red_ t½ or Pc reduction half-time (Pc_red_ t½) should be obtained. By these more precise measures, PCON decreases if ΔpH drops below a threshold value (as observed in *pgr5* and in plants infiltrated with uncouplers) and increases if the cyt*b*_6_*f* ISP is modified. However, in stress conditions such as low CO_2_, some other factor in addition to a threshold ΔpH, is required to induce PCON. Several redox and structure-based mechanisms for this additional regulation of PCON have been proposed and should now be further experimentally tested.

## Materials and methods

Wild-type Col-0 Arabidopsis plants were grown under short-day conditions (9 hours light/15 hours dark) at 22/18°C for 6–8 weeks. Measurements were made with a Dual-KLAS and a Dual-PAM with the P515/535 emitter/detector module (Heinz Walz GmbH) in combination with a Licor 6400 XT (LI-COR Inc.) to allow for control of atmospheric CO_2_. The Licor block temperature was set to 25 °C. For DUAL-KLAS parameters, ETRII was calculated as YII*PAR*0.85, and Y(ND) (P700 oxidation) as (P-P_o_)/P_m_ (Klughammer and Schreiber 1994; Schreiber and Klughammer 2016). The rate constant (K) of P700 re-reduction was calculated by fitting a 1-phase exponential decay function to the data. The P515/535 module was used to measure electrochromic shift as described by Schreiber and Klughammer (2008) and Klughammer et al. (2013). pmf was calculated as the span of a 1-phase exponential decay function (ECSt), normalized to the height of a 50-µs single turnover flash applied before measurements to normalize for leaf thickness and chlorophyll content (Sacksteder and Kramer 2000; Sacksteder et al. 2001). Partitioning of ECSt into ΔpH and Δψ was done according to the method described in Baker et al. (2007). Data analysis, processing, and preparation of plots was done in R Studio using the tidyverse packages (Wickham 2017). Means were compared between CO_2_ conditions at each light intensity using an ordinary 2-way ANOVA and corrected using Tukey multiple comparison test (Supplementary Data Set 1) followed by a HSD test with alpha = 0.05. Different letters indicate significant differences between data points.

## Supplementary Material

koae133_Supplementary_Data

## Data Availability

The data underlying this article will be shared on reasonable request to the corresponding author.

## Supplementary data

The following materials are available in the online version of this article.

**Supplementary Data Set 1**. Statistics for Fig. 4.

## Dive Curated Terms

The following phenotypic, genotypic, and functional terms are of significance to the work described in this paper:

STN7 Gramene: AT1G68830
STN7 Araport: AT1G68830

HCF164 Gramene: AT4G37200
HCF164 Araport: AT4G37200

ATPC1 Gramene: AT4G04640
ATPC1 Araport: AT4G04640

PGR5 Gramene: AT2G05620
PGR5 Araport: AT2G05620
